# Push hard, push fast, if you’re downtown: a citation review of urban-centrism in American and European basic life support guidelines

**DOI:** 10.1186/1757-7241-21-32

**Published:** 2013-04-20

**Authors:** Aaron M Orkin

**Affiliations:** 1 Dalla Lana School of Public Health, University of Toronto, 155 College Street, Toronto, ON M5S 3M2, Canada; 2 Northern Ontario School of Medicine, 955 Oliver Rd, Thunder Bay, ON P7B 5E1, Canada

**Keywords:** Cardiopulmonary resuscitation, Compression-only cardio-pulmonary resuscitation, Bystander cardiopulmonary resuscitation, Basic life support, Rural and remote medicine, Pre-hospital medicine, Guideline creation, Geographic health equity

## Abstract

Bystander cardiopulmonary resuscitation (CPR) improves out-of-hospital cardiac arrest (OHCA) survival. In settings with prolonged ambulance response times, skilled bystanders may be even more crucial. In 2010, American Heart Association (AHA) and European Resuscitation Council (ERC) introduced compression-only CPR as an alternative to conventional bystander CPR under some circumstances. The purpose of this citation review and document analysis is to determine whether the evidentiary basis for 2010 AHA and ERC guidelines attends to settings with prolonged ambulance response times or no formal ambulance dispatch services. Primary and secondary citations referring to epidemiological research comparing adult OHCA survival based on the type of bystander CPR were included in the analysis. Details extracted from the citations included a study description and primary outcome measure, the geographic location in which the study occurred, EMS response times, the role of dispatchers, and main findings and summary statistics regarding rates of survival among patients receiving no CPR, conventional CPR or compression-only CPR. The inclusion criteria were met by 10 studies. 9 studies took place exclusively in urban settings. Ambulance dispatchers played an integral role in 7 studies. The cited studies suggest either no survival benefit or harm arising from compression-only CPR in settings with extended ambulance response times. The evidentiary basis for 2010 AHA and ERC bystander CPR guidelines does not attend to settings without rapid ambulance response times or dispatch services. Standardized bystander CPR guidelines may require adaptation or reconsideration in these settings.

## Introduction

Both high population density and rapid call response times for Emergency Medical Services (EMS) correlate closely with better out-of-hospital cardiac arrest (OHCA) survival rates [1]. Thus, rural populations worldwide face pronounced disparities in OHCA survival rates in comparison with urban populations [2-9]. Bystander resuscitative efforts are known to improve OHCA survival in urban settings [10]. In settings with prolonged call response times, limited or volunteer-based pre-hospital services, or no centralized ambulance dispatch service, skilled bystanders may be all the more crucial for OHCA survival. This underscores the importance of developing bystander CPR guidelines that are well suited for settings with relatively extended EMS call-response times.

Beginning in 2010, basic life support (BLS) guidelines from the American Heart Association (AHA) and European Resuscitation Council (ERC) introduced changes advancing chest compression-only CPR as an alternative in some cases to conventional CPR for untrained and basic responders [11,12] Both organizations advanced these recommendations without specific provisos for settings with prolonged EMS response times or remote geography.

The primary objective of this review is to identify whether the evidentiary basis for compression-only CPR recommendations attend to rural, remote, or geographically underserviced populations. This paper analyses whether the evidence cited by the AHA and ERC included populations with prolonged EMS response times and examines the role of ambulance dispatch services in providing telephone prompts or instructions to OHCA witnesses in the cited studies. This analysis may be used to identify urban-centrism in the cited evidence and guidelines, and to determine whether the evidence cited by 2010 BLS guideline authors can support standard BLS practices in settings with prolonged EMS response times or limited ambulance dispatch services.

### Background – hands-only CPR in the AHA and ERC guidelines

Compression-only (or hands-only) CPR involves the provision of chest compressions to the cardiac arrest patient continuously, without active or direct respiratory support.

**Figure 1:**
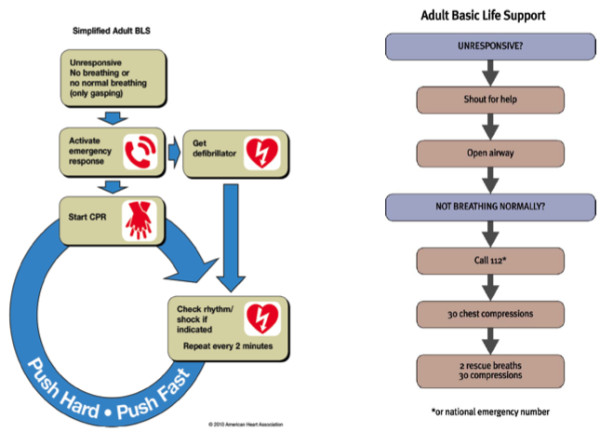
2010 American heart association and European resuscitation council basic life support algorithms.

The 2010 AHA Guidelines encourage hands-only CPR for untrained lay-rescuers and trained lay-rescuers who are not able to perform rescue breaths, indicating that ‘no prospective study of adult cardiac arrest has demonstrated that layperson conventional CPR provides better outcomes than hands-only CPR when provided before EMS arrival’ and that ‘Observational studies of adults with cardiac arrest treated by lay rescuers showed similar survival rates among victims receiving Hands-Only CPR versus conventional CPR with rescue breaths’ [11]. The associated BLS protocol from the AHA Guidelines is provided in Figure 1. The 2010 AHA guidelines emphasized that ‘chest compressions should take priority in the resuscitation of an adult’ and de-emphasized airway and respiratory measures in basic resuscitation, especially for untrained laypeople.

The 2010 ERC guidelines recommend compression-only CPR in a more restrictive fashion to the AHA: ‘laypeople should be encouraged to perform compression-only CPR if they are unable or unwilling to provide rescue breaths, or when instructed during an emergency call to an ambulance dispatcher centre.’ The ERC Guidelines highlight studies of human cardiac arrest suggesting ‘equivalence of chest-compression-only CPR and chest compressions combined with rescue breaths,’ but also emphasizes that no studies ‘excluded the possibility that chest-compression-only is inferior to chest compressions combined with ventilations.’ The ERC concludes that ‘chest compression only will result in insufficient CPR in many cases’. The ERC’s pictorial BLS algorithm includes rescue breaths (Figure 1).

## Methods: citation review and data extraction

### Rationale

In order to better understand how rural populations are reflected in the current guidelines, a citation review and document analysis was performed. This methodology is well established in social science and policy research, and has been identified as an important feature of qualitative research in health sciences [13,14]. This approach is based on the premise that scientific and clinical practice guidelines arise from the evidence cited. Citations represent a literary and scientific acknowledgement that one document receives content from another [13] Since clinical practice guidelines involve the considered interpretation and synthesis of existing scientific literature rather than new primary analyses, guideline authors cite the sources that inform a given recommendation. Therefore, reviewing all cited evidence informing a given guideline should provide support for the guideline itself, and reveals the relationship between the guideline’s assertions and its evidentiary substrate. This methodological approach was used instead of a systematic review deliberately: a systematic review would reveal all available research meeting the desired search criteria, while this study sought to examine only the literature informing the AHA and ERC guidelines.

### Citation search methodology

The citation search was designed to include all primary epidemiological research pertaining to adult survival that was cited by the 2010 AHA and ERC guidelines related to layperson or bystander compression-only CPR in OHCA due to a cardiac cause. Titles and abstracts for all citations included in the 2010 AHA and ERC recommendations related to compression-only CPR were reviewed. The following citations were excluded: human case reports, animal models, mathematical models or simulations, studies in children, studies pertaining primarily to OHCA of non-cardiac aetiology, studies not pertaining to compression-only CPR, and studies of resuscitations performed by healthcare professionals. These citations were excluded because they were not seen to inform epidemiological conclusions regarding human survival in OHCA of cardiac aetiology based on the type of bystander CPR performed. Where citations referred to other syntheses (including systematic reviews, guidelines, narrative reviews or commentaries), a secondary citation search was conducted on these papers according to the same inclusion and exclusion criteria.

### Data abstraction

The following information was abstracted from the full text of the 10 citations included: (1) a study description and primary outcome measure, (2) the geographic location in which the study occurred, (3) available information regarding EMS response time or EMS response time strata, (4) the role of EMS dispatchers or voice assistance in the given study, (5) main findings and summary statistics regarding rates of survival among patients receiving no CPR vs. conventional CPR vs. compression-only CPR. Where multiple papers arose from analyses of the same data set, this information was abstracted from the most recent publication.

## Results

### Citation search

The primary citation search revealed forty citations, composed of 21 cited in the AHA guidelines, and 26 cited in the ERC guidelines. Thirty-one studies were excluded, including 1 case report, 11 studies in animal models, 1 simulation study, 1 study in children, 1 study pertaining to cardiac arrests of non-cardiac aetiology, and 16 studies not pertaining to compression-only CPR survival. Among the 16 citations not pertaining to compression-only CPR survival, the majority (10) attended primarily to attitudes and perceptions among laypeople and professionals to perform resuscitative manoeuvres. This revealed 6 original studies (2 cited by the ERC [15,16], 1 cited by the AHA [17] and 3 cited by both organizations [18-20]) and 3 review citations.

**Figure 2:**
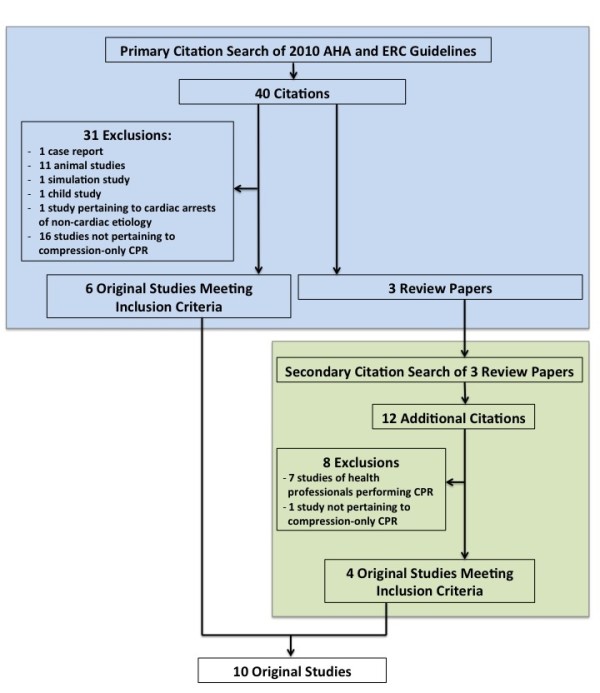
Schematic of citation search.

The 3 review citations were identified as review papers on compression-only CPR or BLS guidelines (2 citations from the AHA [21,22] and 1 citation from the ERC [23]). The structure of these papers permitted immediate identification of citations related to adult OHCA survival based on compression-only CPR vs. other resuscitative manoeuvres, through a published table or literature review. These tables included 12 citations not previously identified through the primary search of AHA and ERC guideline citations. Of the 12 papers cited related to adult OHCA survival based on the type of CPR performed, 7 were excluded because they concerned CPR performed by health professionals and 1 was excluded because it did not pertain to compression-only CPR. The remaining four original studies were included for data abstraction [24-27]. Figure 2 provides a schematic of the citation search process. Additional file 1 provides an annotated version of Figure 2 with a list of all included and excluded citations.

### Citation review

Table 1 provides a summary of data abstracted from the 10 studies meeting inclusion and exclusion criteria. Two studies were found to arise from the same data set, so the most recent publication was selected for data abstraction [24,25].

**Table 1:** Primary and secondary cited OHCA studies in AHA and ERC 2010 guidelines related to adult survival with compression-only CPR

**Citation (year) [citation no.]**	**Source**	**Study description**	**Outcome measure**	**Location**	**Mean EMS response time (minutes) or stratification**	**Role of dispatcher assistance**	**Main finding**	**Bystander CPR by type, rate of survival (%)**		
								**None**	**Comp’n Only CPR**	**Conventional CPR**
**Bohm et al. (2007)**[18]	AHA, ERC, SRS(S1) SRS(S2)	Retrospective cohort study of all patients with OHCA from any cause who received bystander CPR.	1-month survival	Sweden	Not reported. Results stratified into two response time groups <8 vs >8 minutes	No dispatcher support.	No significant difference in outcome between standard CPR and compression-only CPR cohorts. No significant difference identified when cohorts stratified by EMS response time (< 8 min vs. > 8 min).	-	591/8209	77/1145
									(7)	(7)
**Hallstrom (2000)**[26]	SRS(S1) SRS(S2)	RCT of dispatcher instructions for all adult cardiac arrests (toxic causes excluded)	Survival to hospital discharge	King County, Washington USA, (Seattle)	4	Dispatcher randomly assigned to instruct willing bystanders in either compression-only or conventional CPR.	Outcomes with compression alone are similar to outcomes with compressions and mouth-to-mouth ventilation.	-	32/240	29/278
									(15)	(10)
**Iwami et al. (2007)**[19]	AHA, ERC, SRS(S1) SRS(S2)	Prospective population study of all consecutive witnessed adult OHCA patients of presumed cardiac origin.	Neuro. favourable 1-year survival	Osaka, Japan	Results stratified by EMS response time <15 vs. >15 minutes	Not addressed.	Compression-only CPR yielded better outcomes over conventional CPR. For arrests lasting >15 minutes until EMS arrival, neurologically favourable 1-year survival was greater in the conventional CPR group (2.2% vs 0.3%, p < 0.05).	70/2817	19/441	25/617
								(3)	(4)	(4)
**Ong et al. (2008)**[17]	AHA, SRS(S2)	Prospective cohort study of all OHCA patients attended to by EMS providers.	Survival to hospital discharge	Singapore	10.2	During the study period, no dispatcher CPR instructions were given.	No significant difference in outcome between conventional CPR vs. compression-only CPR groups.	9/1695	4/154	8/287
								(0.5)	(2.6)	(2.8)
**Rea et al. (2010)**[15]	ERC	Multicentre RCT of compression-only vs. conventional CPR instruction provided by EMS dispatchers in suspected witnessed OHCA.	Survival to hospital discharge.	King Country and Thurston County, Washington USA and London Ambulance Service, UK.	6.5 (no significant difference between study arms)	Central to study design. All participants received CPR with prompts from EMS dispatcher.	No difference in proportion of patients surviving to hospital discharge by randomization status.	-	122/978 (12.5)	105/956 (11.0)
**SOS-KANTO Study Group (2007)**[20]	AHA, ERC, SRS(S1) SRS(S2)	Prospective cohort study of all witnessed adult cardia arrests of cardiac and noncardiac causes.	Neuro. Favourable 1-month survival	Kanto region, Japan	Results stratified by time from EMS call to first AED analysis ≤10 vs >10 minutes	Dispatcher assistance focused on chest compressions.	Compression-only resuscitation results in better outcome than conventional CPR. No evidence for benefit from mouth-to-mouth ventilation in any subgroup.	63/2917	27/439	30/712
								(2)	(6)	(4)
**Svensson et al. (2010)**[16]	ERC	RCT of compression-only vs. conventional CPR instruction by EMS dispatchers in suspected witnessed OHCA.	30-day survival	Sweden with “inclusion of large rural areas”	Randomization stratified by EMS response time ≤ 5 min, 6–8 min, 9–15 min and >15 min	Central to study design. All participants received CPR with prompts from EMS dispatcher.	No difference with respect to survival at 30 days based on the type of CPR instruction given. Effect consistent across EMS response time strata.	-	54/620 (8.7)	46/656 (7.0)
**Van Hoeyweghen et al. (1993)**[25] Same data set as Bossaert et al. (1989) [24]	SRS(B) SRS(S1) SRS(S2)	Retrospective observational study of all cardiac arrests from all causes, with good quality compression-only or conventional CPR or no CPR.	14-day survival	Belgium	4.3 min in no bystander CPR group, 2.9 minutes in the bystander CPR group	Not addressed.	No statistically significant difference in outcomes in patients who received compression-only CPR vs. conventional CPR.	123/2055	17/116	71/443
								(6)	(15)	(16)
**Waalewijn et al. (2001)**[27]	SRS(S1) SRS(S2)	Prospective observational study of all bystander-witnessed adult cardiac arrests with EMS resuscitation	Survival to hospital discharge	Amsterdam, The Netherlands	Mean not provided. OR of survival 0.83 per minute delay in time to EMS arrival (95% CI 0.76-0.90)	Dispatchers encouraged initiation of ‘basic CPR’, with ventilations.	Similar outcome in cases where chest compression was or was not accompanied by ventilation efforts.	26/429	6/41	61/437
								(6)	(15)	(14)

*AED*, Automated External Defibrillator; *AHA*, American Heart Association; *CI,* confidence interval; *CPR,* cardiopulmonary resuscitation; *EMS,* emergency medical services; *ERC*, European Resuscitation Council; *RCT*, Randomized Controlled Trial; *SRC(B)*, Secondary Review Citation (Becker) [21]; *SRC(S1)*, Secondary Review Citation (Sayre 2008) [22]; *SRC(S2)*, Secondary Review Citation (Sayre 2010) [23]; *UK*: United Kingdom; *USA,* United States of America.

The studies included prospective and retrospective cohort studies, observational studies, and 3 randomized controlled trials. Primary endpoints included a range of survival periods (14-day survival, survival to hospital discharge, 1 month survival) as well as neurologically favourable survival endpoints at 1 month and 1 year (Table 1).

The included studies took place in urban Seattle and Washington State, Singapore, Sweden, Japan, Belgium, the United Kingdom and the Netherlands. No studies were designed or powered to evaluate outcomes in settings with prolonged EMS response times. Mean EMS response times as low as 4 minutes were reported in some studies [26]. Where stratification by call response time occurred, cut-off values for ‘long response time’ (such as 8, 10 or 15 minutes, [19,20]) remained short in comparison with real-world rural or remote conditions. In patients with prolonged bystander resuscitations (>15 minutes), one study demonstrated slightly improved survival in OHCA patients who received conventional CPR instead of hands-only CPR [19]. A randomized controlled trial conducted in Sweden included “large rural areas”, and randomization was stratified by EMS response time to include over 1000 patients with EMS response times >15 minutes [16]. No significant survival difference was seen in this stratum based on the type of bystander CPR performed, but the study was not powered for this sub-group analysis. Two other studies revealed no difference in OHCA outcomes based on the type of bystander resuscitation performed in cases with longer EMS response times (>8 and >10 minutes) [18,20]. While few studies assess the effectiveness of compression-only CPR in settings with a prolonged EMS response time, the cited studies suggest either no survival benefit or harm arising from compression-only CPR in settings with longer EMS response times.

In 7 of the cited studies, EMS dispatchers played an important role or were a central feature of the study’s design and methodology. All three randomized controlled trials were based on randomly assigning dispatchers to prompt OHCA bystanders to perform either conventional or compression-only CPR. Two studies occurred in settings without EMS dispatchers providing CPR prompts or instruction [17,18]. Both were conducted in urban settings with mean EMS response <10 minutes. Both demonstrated no statistically significant difference in survival based on the type of CPR performed.

## Discussion

The 10 papers included in this study form the body of primary epidemiological research on adult OHCA survival with compression-only bystander CPR informing the AHA and ERC 2010 recommendations. These citations reveal biases favouring urban populations in bystander CPR primary research. The strength of this citation review is the ability to uncover and appraise the sources used in the development of dominant resuscitation guidelines.

This approach does face limitations. Citation review assumes that the sources cited by guideline authors (including secondary references in cited review papers) represent all the literature involved in shaping those guidelines, and that guideline authors cite the most important sources involved in guideline development [13]. Few standards exist to ensure that guidelines or other published sources treat citations uniformly. This study should be interpreted as a review of literature informing AHA and/or ERC guidelines, not as a primary systematic review of studies pertaining to compression-only CPR. However, a 2010 systematic review of on compression-only CPR [28] was published after the AHA and ERC guidelines, and revealed one observational study that was not captured by this citation review [29]. This retrospective study found no significant difference in survival to hospital discharge between bystander compression-only and conventional CPR, with a median EMS response time of 9 minutes in both groups. These observations reinforce that compression-only CPR research available to inform 2010 guidelines was specific to settings with relatively short EMS response times.

Two critical observations emerge from these results. First, the cited research examined in this study arises from specific geographic and infrastructural contexts — settings with shorter EMS response times and developed EMS dispatch systems (see Table 1). In contrast, the AHA and ERC guidelines provide universal recommendations for bystander CPR in all settings. Neither organization contextualizes the available evidence within a specific setting or geography where the cited research took place. Both the AHA and ERC recommendations make the explicit assumption that bystander CPR will be performed only briefly with professional services activated or en-route to the patient, and that professional prehospital providers will assume care within minutes [11,12]. These assumptions and their geographical limitations are of critical importance to OHCA witnesses and first responders in settings with prolonged EMS response times. The literature cited by the 2010 AHA and ERC guidelines suggests that compression-only CPR may be no better – and possibly worse – than conventional CPR in improving OHCA survival in settings with prolonged EMS response times.

The second critical observation concerns the confounding role of emergency dispatchers in several of the cited studies (see Table 1). The results of studies where CPR instruction was provided by emergency dispatchers may be less applicable to settings without these services, in isolated or remote settings, or in settings where volunteer responders are telephoned directly, rather than being dispatched by separate personnel.

The literature cited by the AHA and ERC do not appear to support generalized recommendations for universal bystander resuscitation practices irrespective of EMS response times or the role of EMS dispatchers to provide real-time prompting. These observations reinforce concerns regarding ‘knowledge creep and decision accretion’ in medical guideline and policy development, whereby specific research limitations, contexts and uncertainties can become obscured in the development of standard practices [30].

Existing studies establish a clear role for compression-only CPR in bystander resuscitation, especially in settings with rapid EMS response times, and demonstrate other advantages arising from the simplicity of compression-only BLS protocols. Extending the results of urban bystander CPR studies to all settings may not confer survival benefit seen in urban studies, and may disadvantage otherwise underserviced populations disproportionately. Other researchers have explored potential limitations of compression-only CPR guidelines for other special populations [31,32]. The needs of populations in settings with prolonged EMS response times may merit similar attention. The existing data is not adequate to provide robust conclusions regarding compression-only bystander CPR in settings with prolonged EMS response times. Determining the optimal resuscitation practices for these regions will require appropriately powered resuscitation studies conducted in more rural settings. Rural settings may require different BLS recommendations to urban settings.

## Conclusions: geographically specific CPR?

Bystanders and immediate on-scene response is a critical part of the ‘chain of survival’ approach to resuscitation in OHCA [33,34]. In settings with prolonged EMS response or travel times, the resuscitative efforts of bystanders may be even more important to OHCA survival. The evidentiary basis for the AHA and ERC guidelines does not attend to populations with prolonged EMS response times or a lack of formal EMS dispatch services. In some cases, evidence cited by the AHA and ERC suggests a possibility of no benefit [16] or harm [19] when standard compression-only bystander resuscitation guidelines are applied in settings with prolonged EMS response times.

The universal application of 2010 AHA or ERC guidelines may not provide rural and remote responders with the skills or approaches needed to optimize OHCA outcomes in these settings. More research on resuscitations in remote settings is needed to design and understand chains of survival in OHCA in these contexts. Specific attention to these populations in guideline development may also be warranted, especially where the available research displays such a powerful urban-centrism.

Since 2010, the AHA has promoted ‘Push Hard, Push Fast’ as a mantra of bystander CPR. The adage might be aptly refined to ‘Push Hard, Push Fast, If you’re Downtown’. The needs and circumstances of diverse communities and populations may not be met through universal, standardized, and geographically decontextualized approaches to bystander resuscitation or basic life support.

## Abbreviations

AHA: American heart association; AED: Automated external defibrillator; BLS: Basic life support; CPR: Cardiopulmonary resuscitation; EMS: Emergency medical services; ERC: European resuscitation council; OHCA: Out of hospital cardiac arrest; UK: United Kingdom; USA: United States of America.

## Competing interests

No financial or non-financial competing interests to declare.

## Author’s contributions

As the sole author of this paper, I confirm that I meet all of *SJTREM*’s authorship requirements. I was the exclusive contributor to the conception and design of this study, acquisition of data, analysis and data interpretation, drafting the paper, and developing the final version submitted. No other individuals meet these criteria. Individuals who made contributions but do not meet authorship criteria are mentioned in the paper’s acknowledgements.

## Supplementary Material

Additional file 1 Annotated citation search flow chart and list of included and excluded citations. Click here for file
