# Supplementary material for: Push hard, push fast, if you’re downtown: a citation review of urban-centrism in American and European basic life support guidelines
Source: Scand J Trauma Resusc Emerg Med. 2013 Apr 20;21:32. doi: 10.1186/1757-7241-21-32 (PMC3643884; doi:10.1186/1757-7241-21-32)
Supplement: Additional file 1 — Annotated citation search flow chart and list of included and excluded citations. [file 1757-7241-21-32-S1.docx]

# Supplement 1: Annotated Citation Search Flow Chart and List of Included and Excluded Citations


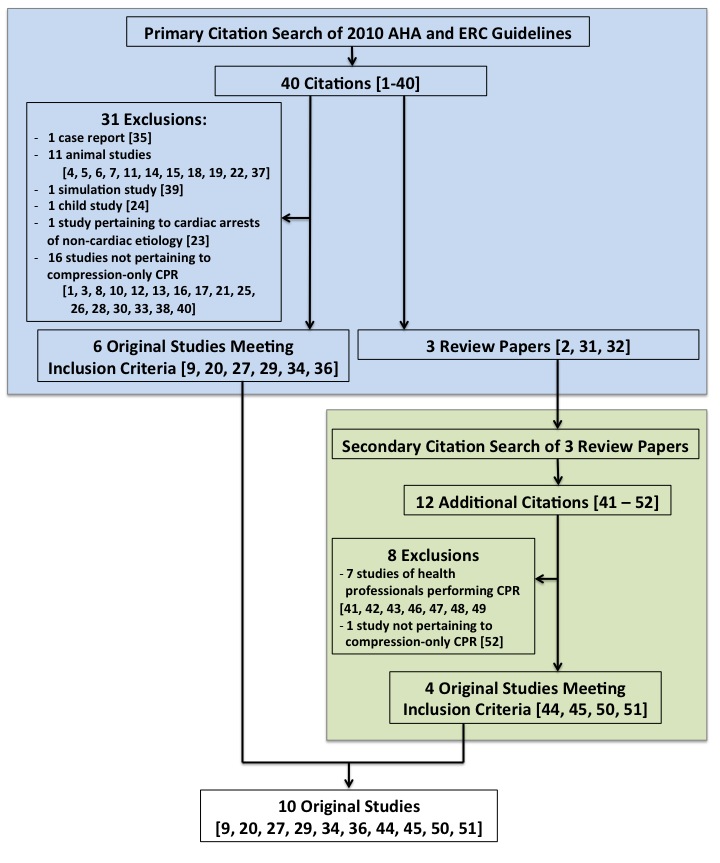


Figure S1: Annotated Citation Search Flow Chart. Bracketed numbers refer to citations in the Table S1 below.

| Table S1: Complete List of Included and Excluded Citations | | | |
| --- | --- | --- | --- |
| Flow Chart Number | Citation | Citation Source | Included/Exclusion [Comments, Exclusion Criterion Where Applicable] |
| 1 | Bång A, Herlitz J, Martinell S. Interaction between emergency medical dispatcher and caller in suspected out-of-hospital cardiac arrest calls with focus on agonal breathing. A review of 100 tape recordings of true cardiac arrest cases. Resuscitation. 2003;56:25-34. | AHA | Excluded [Not about compression-only CPR outcomes] |
| 2 | Becker LB, Berg RA, Pepe PE, et al. A reappraisal of mouth-to-mouth ventilation during bystander-initiated cardiopulmonary resuscitation. A statement for healthcare professionals from the Ventilation Working Group of the Basic Life Support and Pediatric Life Support Subcommittees, American Heart Association. Resuscitation. 1997;35:189-201. | AHA | Included [Review Paper entered into Secondary Search] |
| 3 | Berdowski J, Beekhuis F, Zwinderman AH, Tijssen JGP, Koster RW. Importance of the First Link. Circulation. 2009;119:2096-2102. | AHA | Excluded [Not about compression-only CPR outcomes] |
| 4 | Berg RA, Kern KB, Hilwig RW, et al. Assisted ventilation does not improve outcome in a porcine model of single-rescuer bystander cardiopulmonary resuscitation. Circulation. 1997;95:1635-1641. | AHA, ERC | Excluded [Animal Model] |
| 5 | Berg RA, Kern KB, Hilwig RW, Ewy GA. Assisted ventilation duringbystander'CPR in a swine acute myocardial infarction model does not improve outcome. Circulation. 1997;96:4364-4371. | AHA, ERC | Excluded [Animal Model] |
| 6 | Berg RA, Hilwig RW, Kern KB, Babar I, Ewy GA. Simulated mouth-to-mouth ventilation and chest compressions (bystander cardiopulmonary resuscitation) improves outcome in a swine model of prehospital pediatric asphyxial cardiac arrest. Crit Care Med. 1999;27:1893. | AHA | Excluded [Animal Model] |
| 7 | Berg RA, Hilwig RW, Kern KB, Ewy GA. "Bystander" chest compressions and assisted ventilation independently improve outcome from piglet asphyxial pulseless "cardiac arrest". Circulation. 2000;101:1743-1748. | AHA | Excluded [Animal Model] |
| 8 | Bobrow BJ, Zuercher M, Ewy GA, et al. Gasping during cardiac arrest in humans is frequent and associated with improved survival. Circulation. 2008;118:2550-2554. | AHA, ERC | Excluded [Not about compression-only CPR outcomes] |
| 9 | Bohm K, Rosenqvist M, Herlitz J, Hollenberg J, Svensson L. Survival is similar after standard treatment and chest compression only in out-of-hospital bystander cardiopulmonary resuscitation. Circulation. 2007;116:2908-2912. | AHA, ERC, Sayre 2008, Sayre 2010 | Included |
| 10 | Caves ND, Irwin MG. Attitudes to basic life support among medical students following the 2003 SARS outbreak in Hong Kong. Resuscitation. 2006;68:93-100. | ERC | Excluded [Not about compression-only CPR outcomes] |
| 11 | Chandra NC, Gruben KG, Tsitlik JE, et al. Observations of ventilation during resuscitation in a canine model. Circulation. 1994;90:3070-3075. | ERC | Excluded [Animal Model] |
| 12 | Clark JJ, Larsen MP, Culley LL, Graves JR, Eisenberg MS. Incidence of agonal respirations in sudden cardiac arrest. Ann Emerg Med. 1992;21:1464-1467. | AHA | Excluded [Not about compression-only CPR outcomes] |
| 13 | Donohoe RT, Haefeli K, Moore F. Public perceptions and experiences of myocardial infarction, cardiac arrest and CPR in London. Resuscitation. 2006;71:70-79. | ERC | Excluded [Not about compression-only CPR outcomes] |
| 14 | Dorph E, Wik L, Strømme TA, Eriksen M, Steen PA. Oxygen delivery and return of spontaneous circulation with ventilation:compression ratio 2:30 versus chest compressions only CPR in pigs. Resuscitation. 2004;60:309-318. | ERC | Excluded [Animal Model] |
| 15 | Geddes LA, Rundell A, Otlewski M, Pargett M. How much lung ventilation is obtained with only chest-compression CPR? Cardiovasc Eng. 2008;8:145-148. | ERC | Excluded [Animal Model] |
| 16 | Hew P, Brenner B, Kaufman J. Reluctance of paramedics and emergency medical technicians to perform mouth-to-mouth resuscitation. J Emerg Med. 1997;15:279-284. | ERC | Excluded [Not about compression-only CPR outcomes] |
| 17 | Hubble MW, Bachman M, Price R, Martin N, Huie D. Willingness of high school students to perform cardiopulmonary resuscitation and automated external defibrillation. Prehosp Emerg Care. 2003;7:219-224. | ERC | Excluded [Not about compression-only CPR outcomes] |
| 18 | Idris AH, Becker LB, Fuerst RS, et al. Effect of ventilation on resuscitation in an animal model of cardiac arrest. Circulation. 1994;90:3063-3069. | AHA | Excluded [Animal Model] |
| 19 | Iglesias JM, López-Herce J, Urbano J, Solana MJ, Mencía S, del Castillo J. Chest compressions versus ventilation plus chest compressions in a pediatric asphyxial cardiac arrest animal model. Intensive Care Med. 2010;36:712-716. | AHA | Excluded [Animal Model] |
| 20 | Iwami T, Kawamura T, Hiraide A, et al. Effectiveness of bystander-initiated cardiac-only resuscitation for patients with out-of-hospital cardiac arrest. Circulation. 2007;116:2900-2907. | AHA, ERC, Sayre 2008, Sayre 2010 | Included |
| 21 | Jelinek GA, Gennat H, Celenza T, O'Brien D, Jacobs I, Lynch D. Community attitudes towards performing cardiopulmonary resuscitation in Western Australia. Resuscitation. 2001;51:239-246. | ERC | Excluded [Not about compression-only CPR outcomes] |
| 22 | Kern KB, Hilwig RW, Berg RA, Sanders AB, Ewy GA. Importance of continuous chest compressions during cardiopulmonary resuscitation: improved outcome during a simulated single lay-rescuer scenario. Circulation. 2002;105:645-649. | ERC | Excluded [Animal Model] |
| 23 | Kitamura T, Iwami T, Kawamura T, et al. Bystander-initiated rescue breathing for out-of-hospital cardiac arrests of noncardiac origin. Circulation. 2010;122:293-299. | ERC | Excluded [Cardiac arrest of non-cardiac etiology] |
| 24 | Kitamura T, Iwami T, Kawamura T, et al. Conventional and chest-compression-only cardiopulmonary resuscitation by bystanders for children who have out-of-hospital cardiac arrests: a prospective, nationwide, population-based cohort study. Lancet. 2010;375:1347-1354. | AHA, ERC | Excluded [Pediatrics] |
| 25 | Lam KK, Lau FL, Chan WK, Wong WN. Effect of severe acute respiratory syndrome on bystander willingness to perform cardiopulmonary resuscitation (CPR)--is compression-only preferred to standard CPR? Prehosp Disaster Med. 2007;22:325-329. | ERC | Excluded [Not about compression-only CPR outcomes] |
| 26 | Locke CJ, Berg RA, Sanders AB, et al. Bystander cardiopulmonary resuscitation. Concerns about mouth-to-mouth contact. Arch Intern Med. 1995;155:938-943. | ERC | Excluded [Not about compression-only CPR outcomes] |
| 27 | Ong MEH, Ng FSP, Anushia P, et al. Comparison of chest compression only and standard cardiopulmonary resuscitation for out-of-hospital cardiac arrest in Singapore. Resuscitation. 2008;78:119-126. | AHA, Sayre 2010 | Included |
| 28 | Ornato JP, Hallagan LF, McMahan SB, Peeples EH, Rostafinski AG. Attitudes of BCLS instructors about mouth-to-mouth resuscitation during the AIDS epidemic. Ann Emerg Med. 1990;19:151-156. | ERC | Excluded [Not about compression-only CPR outcomes] |
| 29 | Rea TD, Fahrenbruch C, Culley L, et al. CPR with chest compression alone or with rescue breathing. New England Journal of Medicine. 2010;363:423-433. | ERC | Included |
| 30 | Sanders AB, Otto CW, Kern KB, Rogers JN, Perrault P, Ewy GA. Acid-base balance in a canine model of cardiac arrest. Ann Emerg Med. 1988;17:667-671. | AHA | Excluded [Not about compression-only CPR outcomes] |
| 31 | Sayre MR, Berg RA, Cave DM, Page RL, Potts J, White RD. Hands-only (compression-only) cardiopulmonary resuscitation: A call to action for bystander response to adults who experience out-of-hospital sudden cardiac arrest. Circulation. 2008;117:2162-2167. | AHA | Included [Review Paper entered into Secondary Search] |
| 32 | Sayre MR, Koster RW, Botha M, et al. Part 5: Adult basic life support: 2010 International Consensus on Cardiopulmonary Resuscitation and Emergency Cardiovascular Care Science With Treatment Recommendations. Circulation. 2010;122:S298-S324. | ERC | Included [Review Paper entered into Secondary Search] |
| 33 | Shibata K, Taniguchi T, Yoshida M, Yamamoto K. Obstacles to bystander cardiopulmonary resuscitation in Japan. Resuscitation. 2000;44:187-193. | ERC | Excluded [Not about compression-only CPR outcomes] |
| 34 | SOS-KANTO Study Group. Cardiopulmonary resuscitation by bystanders with chest compression only (SOS-KANTO): an observational study. The Lancet. 2007;369. | AHA, ERC, Sayre 2008, Sayre 2010 | Included |
| 35 | Steen-Hansen JE. Favourable outcome after 26 minutes of "Compression only" resuscitation: a case report. Scand J Trauma Resusc Emerg Med. 2010;18:19. | AHA | Excluded [Case Report] |
| 36 | Svensson L, Bohm K, Castrèn M, et al. Compression-only CPR or standard CPR in out-of-hospital cardiac arrest. New England Journal of Medicine. 2010;363:434-442. | ERC | Included |
| 37 | Tang W, Weil MH, Sun S, et al. Cardiopulmonary resuscitation by precordial compression but without mechanical ventilation. American journal of respiratory and critical care medicine. 1994;150:1709-1713. | AHA | Excluded [Animal Model] |
| 38 | Taniguchi T, Omi W, Inaba H. Attitudes toward the performance of bystander cardiopulmonary resuscitation in Japan. Resuscitation. 2007;75:82-87. | ERC | Excluded [Not about compression-only CPR outcomes] |
| 39 | Turner I, Turner S, Armstrong V. Does the compression to ventilation ratio affect the quality of CPR: a simulation study. Resuscitation. 2002;52:55-62. | ERC, Sayre 2010 | Simulation/Model |
| 40 | Weil MH, Rackow EC, Trevino R, Grundler W, Falk JL, Griffel MI. Difference in acid-base state between venous and arterial blood during cardiopulmonary resuscitation. N Engl J Med. 1986;315:153-156. | AHA | Excluded [Not about compression-only CPR outcomes] |
| 41 | Bertrand C, Hemery F, Carli P, et al. Constant flow insufflation of oxygen as the sole mode of ventilation during out-of-hospital cardiac arrest. Intensive Care Med. 2006;32:843-851. | Sayre 2010 | Excluded [Professional Providers] |
| 42 | Bobrow BJ, Clark LL, Ewy GA, et al. Minimally interrupted cardiac resuscitation by emergency medical services for out-of-hospital cardiac arrest. JAMA. 2008;299:1158-1165. | Sayre 2010 | Excluded [Professional Providers] |
| 43 | Bobrow BJ, Ewy GA, Clark L, et al. Passive oxygen insufflation is superior to bag-valve-mask ventilation for witnessed ventricular fibrillation out-of-hospital cardiac arrest. Ann Emerg Med. 2009;54:656-662.e1. | Sayre 2010 | Excluded [Professional Providers] |
| 44 | Bossaert L, Van Hoeyweghen R. Bystander cardiopulmonary resuscitation (CPR) in out-of-hospital cardiac arrest. Resuscitation. 1989;17:S55-S69. | Becker, Sayre 2008 |  |
| 45 | Hallstrom A, Cobb L, Johnson E, Copass M. Cardiopulmonary resuscitation by chest compression alone or with mouth-to-mouth ventilation. New England Journal of Medicine. 2000;342:1546-1553. | Sayre 2010, Sayre 2008 | Included |
| 46 | Kellum MJ, Kennedy KW, Ewy GA. Cardiocerebral resuscitation improves survival of patients with out-of-hospital cardiac arrest. Am J Med. 2006;119:335-340. | Sayre 2010 | Excluded [Professional Providers] |
| 47 | Kellum MJ, Kennedy KW, Barney R, et al. Cardiocerebral resuscitation improves neurologically intact survival of patients with out-of-hospital cardiac arrest. Ann Emerg Med. 2008;52:244-252. | Sayre 2010 | Excluded [Professional Providers] |
| 48 | Krischer JP, Fine EG, Weisfeldt ML, Guerci AD, Nagel E, Chandra N. Comparison of prehospital conventional and simultaneous compression-ventilation cardiopulmonary resuscitation. Crit Care Med. 1989;17:1263-1269. | Sayre 2010 | Excluded [Professional Providers] |
| 49 | Saïssy JM, Boussignac G, Cheptel E, et al. Efficacy of continuous insufflation of oxygen combined with active cardiac compression-decompression during out-of-hospital cardiorespiratory arrest. Anesthesiology. 2000;92:1523-1530. | Sayre 2010 | Excluded [Professional Providers] |
| 50 | Van Hoeyweghen RJ, Bossaert LL, Mullie A, et al. Quality and efficiency of bystander CPR. Resuscitation. 1993;26:47-52. | Becker, Sayre 2008, Sayre 2010 | Included |
| 51 | Waalewijn RA, Tijssen JGP, Koster RW. Bystander initiated actions in out-of-hospital cardiopulmonary resuscitation: results from the Amsterdam Resuscitation Study (ARRESUST). Resuscitation. 2001;50:273-279. | Sayre 2010, Sayre 2008 | Excluded [Not about compression-only CPR outcomes] |
| 52 | Wik L, Steen PA, Bircher NG. Quality of bystander cardiopulmonary resuscitation influences outcome after prehospital cardiac arrest. Resuscitation. 1994;28:195-203. | Sayre 2010 | Included |
